# Anesthetic management of lung transplantation in a patient with end-stage COVID-19 pneumonia: A case report

**DOI:** 10.1097/MD.0000000000026468

**Published:** 2021-06-25

**Authors:** Shao-Hui Guo, Ang Li, Peng-Fei Yin, Sheng-Mei Zhu, Yong-Xing Yao

**Affiliations:** Department of Anaesthesia, First Affiliated Hospital, Zhejiang University School of Medicine, Hangzhou, P. R. China.

**Keywords:** anaesthesia, COVID-19, extracorporeal membrane oxygenation, lung transplantation, pneumonia

## Abstract

**Rationale:**

The COVID-19 pandemic is spreading around the world and the leading cause of death is rapidly progressive respiratory failure because of lung damage and consolidation. Lung transplantation is the last line of treatment for chronic end-stage lung diseases. There were several cases of lung transplantation reported in patients with COVID-19 pneumonia. However, anesthetic management of lung transplantation in this subpopulation is rare. We report the anesthetic and perioperative management of lung transplantation in a patient with COVID-19 pneumonia.

**Patient concerns:**

A 70-year-old man with a 7-day history of fever was diagnosed with COVID-19 pneumonia. His throat swab was positive for COVID-19, but negative for other common viruses. Chest radiography showed multiple inflammatory foci in both lungs. By day 5, he presented respiratory distress. Computed tomography (CT) scan showed progressive deterioration of both lungs. Starting on day 7, SARS-CoV-2 RNA in bronchoalveolar lavage samples were continuously negative. However, his lung condition deteriorated. By day 17, a veno-venous extracorporeal membrane oxygenation (ECMO) was initiated. After 10 days of ECMO support, the patient's lung condition did not improve. CT scan revealed bilateral parenchymal consolidation with pulmonary fibrosis and hydrothorax.

**Diagnosis:**

Irreversible lung function loss induced by COVID-19 pneumonia.

**Interventions:**

Bilateral transplantation was performed because the patient's lung condition did not improve and CT scan revealed parenchymal consolidation with pulmonary fibrosis after 10 days of ECMO support. Thirty-six hours after the surgery, ECMO was discontinued. A percutaneous transluminal coronary angioplasty and a stent implantation were performed because of acute coronary syndrome and myocardial ischemia 4 days postoperatively.

**Outcomes:**

The patient remained hospitalized because of requirements for intermittent assisted ventilation via tracheostomy.

**Lessons:**

This case further supports the consideration that lung transplantation can potentially be the successful therapy for these patients who have developed irreversible lung function lose due to COVID-19 pneumonia. However, most critical patients with COVID-19 are older individuals with various comorbidities, which present new anesthetic challenges.

## 1 Introduction

At present, nearly 30 million people have been infected worldwide with severe acute respiratory syndrome coronavirus 2 (SARS-CoV-2, which causes coronavirus disease [COVID-19]). Despite intensive medical therapy, the case fatality rate remains 5.7%.^[1]^ The leading cause of death in patients with COVID-19 is rapidly progressive respiratory failure because of lung damage and consolidation.^[2,3]^ Lung transplantation is considered the last line of treatment for end-stage lung diseases. There were several successful cases of lung transplantation in patients with COVID-19 pneumonia.^[4–6]^ However, anesthetic management of lung transplantation in this subpopulation has not been reported so far. In this case report, we focus on the anesthetic and perioperative management of urgent lung transplantation in a patient with COVID-19 pneumonia-induced irreversible lung function loss.

## 2 Case presentation

**Figure 1:**
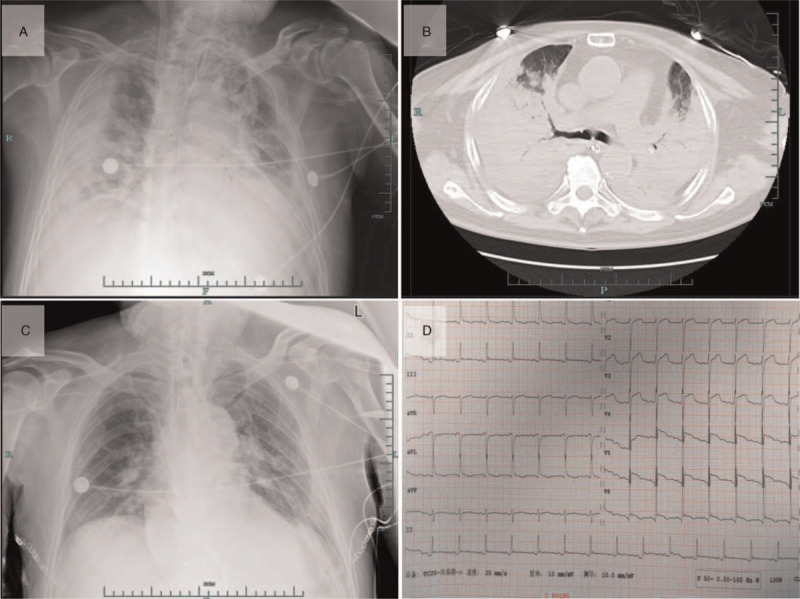
(A) Chest radiograph acquired at admission showing multiple inflammatory foci in both lungs. (B) Chest computed tomography scan performed on day 26 showing severe parenchymal consolidation with bilateral pulmonary fibrosis and hydrothorax. (C) Chest radiograph acquired on the day following transplantation showing scattered inflammation in the bilateral lung fields. (D) Electrocardiogram revealing ST elevation on the continuous chest lead.

The Ethical Committee of the Zhejiang University School of Medicine approved this study and written patient consent was obtained for publication of this case. A 70-year-old man with a 7-day history of fever was hospitalized on 9 February 2020, and diagnosed with COVID-19 pneumonia. He had a positive medical history of diabetes for 10 years and hypertension for 5 years. On admission, his temperature was 37.8°C, heart rate 82 beats/min^1^, respiratory rate 22 breaths/min, blood pressure (BP) 141/78 mmHg, percutaneous oxygen saturation 96% (fraction of inspiration oxygen [*F*_I_O_2_] 0.5). Laboratory test results showed a C-reactive protein concentration of 433.1 nmol/L (45.48 mg/L), with normal coagulation profile and serum enzyme levels. Complete blood count showed elevated leukocytes (13 × 10^9^ cells/L), and neutrophils (92.3%), but low lymphocyte count (0.5 × 10^9^ cells/L). The patient's throat swab was positive for COVID-19, but negative for other common viruses. Chest radiography showed multiple inflammatory foci in both lungs (Fig. 1A).

He was transferred to the ICU and antibiotics (arbidol, 0.2 g every 8 hours; tazocin, 4.5 g every 8 hours) and recombinant human interferon alfa-1b were administered via aerosol 6hourly. By day 5, he presented respiratory distress (35–40 breath/min). Chest computed tomography (CT) scan showed progressive deterioration of both lungs. On blood gas analysis, partial pressure of carbon dioxide (*P*_a_CO_2_) was 41 mmHg, and partial pressure of oxygen was 46 mmHg. Tracheal intubation was performed and pressure ventilation mode (*F*_I_O_2_ 0.55, positive end-expiratory pressure 10 cm H_2_O, peak pressure 25–30 cm H_2_O) was applied. Starting on day 7, SARS-CoV-2 RNA in bronchoalveolar lavage samples were continuously negative. However, his lung condition deteriorated. By day 17, blood gas analysis showed a *P*_a_CO_2_ of 44 mmHg, and partial pressure of oxygen 61 mmHg (*F*_I_O_2_ 0.75, peak pressure 30 cm H_2_O). Veno-venous extracorporeal membrane oxygenation (V-V extracorporeal membrane oxygenation [ECMO], BE-PLS 2050, MAQUET, Germany) with dual cannulae from the right femoral vein and right internal jugular vein was established (target flow rate 3.5 L/min) to improve oxygenation (Table 1 shows the timeline).

**Table 1:** Chronology of major events.

Events	Time (2020)	Note/Management
Hospital admission (ICU)	February 9	Fever, SARS-CoV-2 (+), diabetes, hypertension.
Mechanical ventilation	February 14	PC mode (25–30 cm H_2_O).
Viral tests negative	February 16	BAL (−), faeces (−).
Veno-venous ECMO	February 26	Femoral and internal jugular vein.
Lung transplantation	March 8	Venous-arterial ECMO.
Weaned from ECMO	March 11	PC mode (18 cm H_2_O).
Arrhythmia, hypotension	March 12	PTCA + stent implantation.

BAL = bronchoalveolar lavage, ECMO = extracorporeal membrane oxygenation, PTCA = percutaneous transluminal coronary angioplasty, SARS-CoV-2 = severe acute respiratory syndrome coronavirus 2.

After 10 days of ECMO support, the patient's lung condition did not improve, with extremely low compliance and without effective ventilation (<100 mL tidal volume under 30 cm H_2_O pressure). CT scan revealed bilateral parenchymal consolidation with pulmonary fibrosis and hydrothorax (Fig. 1B). A multidisciplinary team of specialists from the ICU, infectious diseases, respiratory, surgery, and anesthesiology departments was consulted. Lung transplantation was the last resort to maintain his life. After Institutional Ethical Committee approval, the patient was registered in the China Organ Transplant Response System. He was matched to a lung donor with brain death, and a transplantation was arranged.

Before induction, low-dose dobutamine (2 μg/kg/min) was infused and arterial cannulation was established for continuous BP monitoring. General anesthesia was induced with midazolam 3 mg, propofol 50 mg and sufentanil 30 μg, rocuronium 50 mg was administered, and anaesthesia was maintained with sevoflurane 1.2%, remifentanil 0.25 mg/h and cisatracurium 10 mg/h) in 80% oxygen to maintain stable hemodynamics. Mechanical ventilation with pressure-controlled mode (30 cm H_2_O, tidal volume 50–80 mL) was applied. ECMO was adjusted to keep *P*_a_CO_2_ within the range of 35 to 40 mmHg. Preoperative transoesophageal echocardiography showed a normal heart size and cardiac function (left ventricular ejection fraction [LVEF] 55%–60%); pulmonary arterial systolic BP was estimated to be 35 mmHg.

In addition to standard monitoring, blood gas and hemoglobin analyses were performed hourly to maintain a stable inner milieu. The procedure was performed with a clamshell incision. Intraoperative extracorporeal life support was changed from V-V ECMO to a standalone veno-arterial (V-A) ECMO via central cannulation to provide cardiopulmonary support and attenuate the ischemic–reperfusion injury during implantation. The right and left lungs were transplanted successively. The right middle lobe was removed because of its larger size. The operation was uneventful, lasted for 3 hours. On left lung resection using a pulmonary vein clamp and left atrial stretching, ventricular fibrillation occurred abruptly. After intravenous lidocaine 1.5 mg/kg and electrical defibrillation (20 J twice), sinus rhythm was restored. Contractility and hemodynamics were stabilized with infusions of dobutamine 1 μg/kg/min and noradrenaline 2 μg/min.

The cold ischemic time of the donor lung was 8 hours. After reperfusion, the compliance improved immediately. Packed red cells (3720 mL) and fresh frozen plasma (2380 mL) were infused intraoperatively. Postoperatively, the central V-A ECMO was converted to a peripheral V-V ECMO for postoperative continuation. Mechanical ventilation (pressure controlled mode, *F*_I_O_2_ 0.4, positive end-expiratory pressure 8 cm H_2_O, PC 18 cm H_2_O, tidal volume 500 mL) was applied. Triple immunosuppression therapy was started postoperatively with tacrolimus, mycophenolate (CellCept), and glucocorticoids. The patient's lung function and compliance improved with subsequent discontinuation of V-A ECMO 36 hours postoperatively. Chest radiograph showed scattered bilateral inflammation (Fig. 1C). The primary graft dysfunction grades at 0, 24, and 72 hours were ungradable, ungradable, and 0, respectively. Histopathological examination of the resected lung specimens revealed extensive consolidation, diffuse small airway injury with partial alveolar hemorrhage and features characteristic of end-stage viral pneumonia. Multiple site analyses and virus culture were negative.

On postoperative day 4, repeated episodes of supraventricular and ventricular tachycardia and hypotension were detected. ECG showed ST-segment elevation (Fig. 1D). Echocardiography findings indicated decreased movement in the ventricular septum and anterior wall. Acute coronary syndrome and myocardial ischemia were considered because of the unstable hemodynamics and high cardiac troponin I levels. Subsequent percutaneous coronary intervention found severe stenosis in the proximal segment of the left anterior descending branch and mild to-moderate lesions in the remaining coronary arteries. A percutaneous transluminal coronary angioplasty of the left anterior descending branch with a drug-eluting stent implantation was performed; postoperatively, thrombolysis in myocardial infarction grade 3 blood flow was detected. Postoperatively, clopidogrel and aspirin were administered as anticoagulants. Dobutamine and noradrenaline were discontinued on postoperative days 7 and 9, respectively. The patient's cardiac condition stabilized with therapy, but he remained hospitalized because of requirements for intermittent assisted ventilation via tracheostomy.

## 3 Discussion

Since the first successful report by James Hardy in 1963, lung transplantation has become a widely accepted treatment for patients with end-stage lung diseases.^[7]^ Except for chronic obstructive pulmonary diseases, interstitial lung disease, cystic fibrosis, and pulmonary arterial hypertension, indications for lung transplantation have changed in recent years, especially during the COVID-19 pandemic.^[4,7]^ In our patient, after 27 days of intensive therapy (including 22 days of mechanical ventilation and 10 days of ECMO support), both lungs deteriorated to irreversible parenchymal consolidation with established pulmonary fibrosis; therefore, lung transplantation was considered the ultimate rescue treatment. As a highly infectious disease, COVID-19 pneumonia is fundamentally different from other chronic lung diseases, and the profile of the disease is largely unknown. Anesthetic management in lung transplantation in patients with COVID-19 presents extraordinary challenges.^[8]^ In this case, the procedure was performed in an operating room located at the corner of the operating complex, with negative pressure environment and separate access to minimize infection risk. All anesthesia equipment was kept in places that satisfied the hygienic requirements for preventing nosocomial infections. Standard procedures for personnel protection were re-examined by antiepidemic experts as described previously.^[9]^

Most critical patients with COVID-19 are older individuals with various comorbidities (diabetes mellitus, hypertension and coronary artery disease), which present new anesthetic challenges.^[10]^ In this case, the surgical manoeuvre-induced intraoperative ventricular fibrillation may have occurred because of an unidentified coronary artery disease, consistent with the postoperative arrhythmias, subsequent ECG and coronary angiography findings. However, ventricular fibrillation can have multiple causes. Timely detection and effective management prevented the patient's condition from progressing to severe myocardial infarction and cardiac arrest.

## 4 Conclusions

Undoubtedly, the COVID-19 pandemic is the most difficult challenge for the human beings in recent decades. This case report further supports the consideration that lung transplantation can potentially be the successful therapy for these patients who have developed irreversible lung function lose due to COVID-19. However, the long-term outcomes of lung transplantation in these patients need to be verified in future. As an indispensable member of the multidisciplinary transplant team, anesthesiologists play a vital role in the present COVID-19 pandemic.

## Acknowledgment

The authors thank Maxwell R. Shen for English language editing.

## Author contributions

**Conceptualization & writing:** Yong-Xing Yao.

**Conceptualization:** Sheng-Mei Zhu, Yong-Xing Yao.

**Data curation:** Shao-Hui Guo and Peng-Fei Yin, Ang Li.

**Writing – original draft:** Ang Li, Shao-Hui Guo.

**Writing – review & editing:** Ang Li, Peng-Fei Yin, Sheng-Mei Zhu, Yong-Xing Yao.
